# Computational Studies on Optoelectronic and Nonlinear Properties of Octaphyrin Derivatives

**DOI:** 10.3389/fchem.2017.00011

**Published:** 2017-03-06

**Authors:** Nasarul Islam, Irfan H. Lone

**Affiliations:** ^1^Department of Chemistry, Guru Nanak Dev UniversityAmritsar, India; ^2^Department of Chemistry, Government Degree CollegeKupwara, India

**Keywords:** porphyrin, reorganization energy, bond-length-alternation, mesomeric effect, polarizability, hyperpolarizability

## Abstract

The electronic and nonlinear optical (NLO) properties of octaphyrin derivatives were studied by employing the DFT/TDFT at CAM-B3LYP/6-311++G (2d, 2p) level of the theory. Thiophene, phenyl, methyl and cyano moieties were substituted on the molecular framework of octaphyrin core, in order to observe the change in optoelectronic and nonlinear response of these systems. The frontier molecular orbital studies and values of electron affinity reveals that the studied compounds are stable against the oxygen and moisture present in air. The calculated ionization energies, adiabatic electron affinity and reorganization energy values indicate that octaphyrin derivatives can be employed as effective n-type material for Organic Light Emitting Diodes (OLEDs). This character shows an enhancement with the introduction of an electron withdrawing group in the octaphyrin framework. The polarizability and hyperpolarizability values of octaphyrin derivatives demonstrate that they are good candidates for NLO devices. The nonlinear response of these systems shows enhancement on the introduction of electron donating groups on octaphyrin moiety. However, these claims needs further experimental verification.

## Introduction

The discovery of sapphyrins and expanded porphyrins have attracted the interest of researchers attributed to their diverse applications in materials science (Sprutta and Latos-Grazyński, 2001; Flemming and Dolphin, 2002; Pushpan et al., 2002; Silva et al., 2002a,b; Cavaleiro et al., 2003; Hata et al., 2006; Tanaka and Osuka, 2016). The defining feature of these macrocycles is the presence of a larger internal cavity as compared to those present in natural tetrapyrroles. More specifically, expanded porphyrins are macrocyclic compounds containing five-membered heterocyclic units (like pyrrole, furan, or thiophene) linked together, either directly or through spacers with internal ring pathway contains at least 17 atoms (Sprutta and Latos-Grazyński, 2001; Flemming and Dolphin, 2002; Pushpan et al., 2002; Silva et al., 2002a,b; Hata et al., 2006). Their distinctive physical and structural properties have found applications in nonlinear optical (NLO) materials (Marder et al., 1997; Zhou et al., 2002; Ahn et al., 2005; Rath et al., 2005a; Misra et al., 2006), photosensitizers for photodynamic therapy (PDT) (Harriman et al., 1989; Maiya et al., 1989), transition or rare earth metal ion chelates, cation and anion receptors (Shionoya et al., 1992; Jasat and Dolphin, 1997; Sessler et al., 2000a,b; Sessler and Davis, 2001; Chandrashekar and Venkatraman, 2003), magnetic resonance imaging (MRI) contrasting agents (Charrière et al., 1993; Weghorn et al., 1996; Werner et al., 1999) and a tool for accessing presently unknown higher aromatic systems (Sessler et al., 1993). These fascinating properties have inspired synthetic efforts toward a range of expanded porphyrins differing in ring size, ring connectivity, peripheral substituent and core modification (Hiroto et al., 2006; Misra and Chandrashekar, 2008; Anaka et al., 2011; Mori et al., 2012a; Kido et al., 2013; Naoda and Osuka, 2014; Anguera et al., 2015). These modifications not solely amendment their electronic properties, but also conjointly create structural diversity to induce ring inversion in the resulting macrocycles. Currently, considerable attention has been focused on the studies of organic molecules capable of exhibiting organic light emitting properties or massive NLO susceptibilities. Researchers have observed that the presence of extended π-electron delocalization is the key element in the design of organic molecules exhibiting either the OLED or NLO applications (Geffroy et al., 2006; Sasabe and Kido, 2011, 2013a,b; Jin and Tang, 2013; Sekine et al., 2014; Islam and Pandith, 2014a; Romain et al., 2015).

**Figure:**
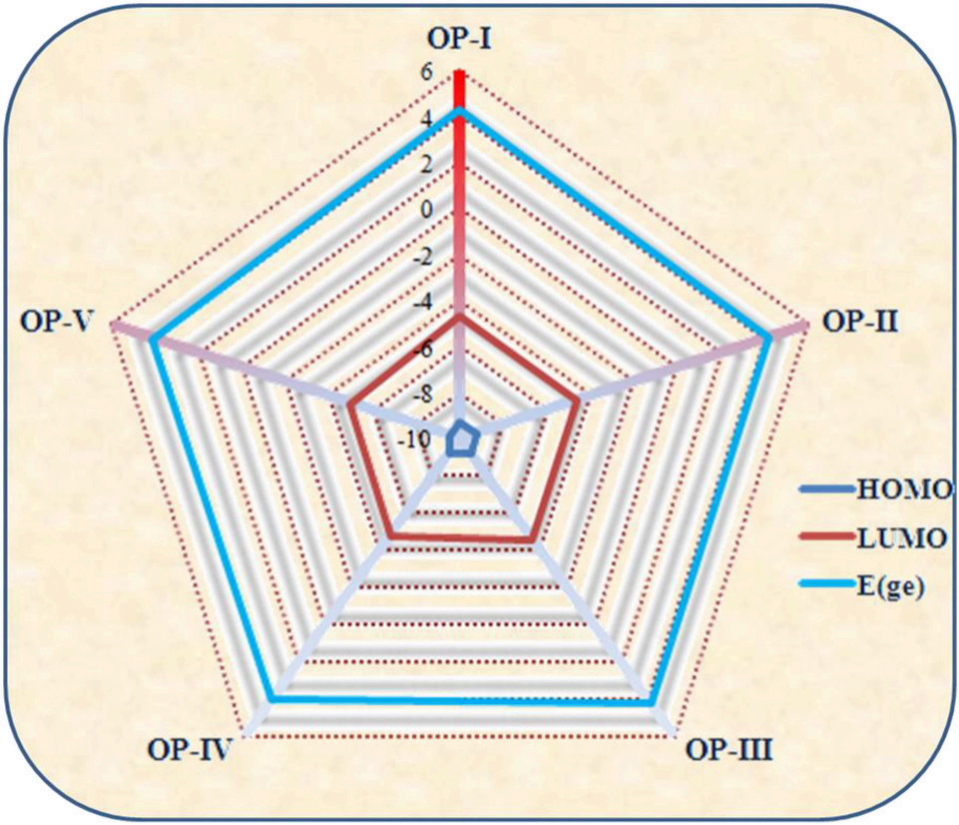
**Graphical Abstract**.

The expanded porphyrins that display the greatest resemblance to natural porphyrins are those containing either meso-like bridging carbon atoms or direct links between the heterocyclic subunits. According to the nomenclature put forward by Franck and Nonn (Franck and Nonn, 1995), the name of these systems consists of three parts: (1) the number of π-electrons in the shortest conjugation pathway (in square brackets), (2) a core name indicating the number of pyrroles or other heterocycles (e.g., pentaphyrin, hexaphyrin), and (3) the number of bridging carbon atoms between each pyrrole subunit (in round brackets and separated by dots). For instance, according to this nomenclature, the classic porphyrin macrocycle presented in the heme group would be named as [18] tetraphyrin (1.1.1.1). Octaphyrin (1.1.1.1.1.1.0.0) (**OP**), being a conjugated π system may be the excellent candidate for the materials science. Many research groups have reported the synthesis and structural properties of isomers of Octaphyrin (1.1.1.1.1.1.0.0) (Franck and Nonn, 1995; Latos-Grazynski, 2004; Shimizu et al., 2005; Rath et al., 2005b; Geffroy et al., 2006; Hiroto et al., 2006; Kumar et al., 2007; Misra and Chandrashekar, 2008; Anaka et al., 2011; Sasabe and Kido, 2011, 2013a,b; Mori et al., 2012a; Jin and Tang, 2013; Kido et al., 2013; Naoda and Osuka, 2014; Sekine et al., 2014; Islam and Pandith, 2014a; Anguera et al., 2015; Romain et al., 2015). According to Chandershaker et al. the core-modified expanded porphyrins containing 26, 36, and 54 π electrons because of their exceptionally massive two-photon absorption cross- sections may be considered among the most effective appropriate candidates, particularly as organic NLO materials (Pushpan and Chandrashekar, 2002). The derivatives of hexaphyrins and octaphyrins containing meso-imidazolyl groups were prepared by research group of Hirotaka et al. (Mori et al., 2012b). They found that hydrogen bonding is effective for the development of Huckel antiaromatic expanded porphyrins. The molecular framework of octaphyrin is consistent with a 36 π-electron circuit within what can be considered a twisted double-side (orientable) Huckel topology (Sprutta and Latos-Grazyński, 2001; Flemming and Dolphin, 2002; Pushpan et al., 2002; Silva et al., 2002a,b; Hata et al., 2006). According to Osuka et al. the hydrogens present near the crossing point of the octaphyrin resonate at δ = 17.14 and 8.60 ppm in the deshielding region. These findings are consistent with the presence of paratropic ring current and categorizing the octaphyrin among the Huckel type antiaromatic system. Various studies were performed for explaining the conformational switch between Hückel planar and Möbius twisted topologies of expanded porphyrins (Torrent-Sucarrat et al., 2012; Alonso et al., 2013, 2014; Marcos et al., 2014). Observations revealed that the nature of the meso-substituent is important for determining the relative stability of the Hückel−Möbius conformers and interconversion also between them is controlled by the barrier height (Torrent-Sucarrat et al., 2012; Marcos et al., 2014). Proft et al. employed DFT at B3LYP/6-31G (d,p) level of theory to study the conformational preferences, interconversion pathways and aromaticity of N-fused [22] and [24] pentaphyrins. They have observed that the choice of conformation strongly depend upon the oxidation state, aromaticity of the π-electron system and meso-substituents (Alonso et al., 2013, 2014).

**Figure 1:**
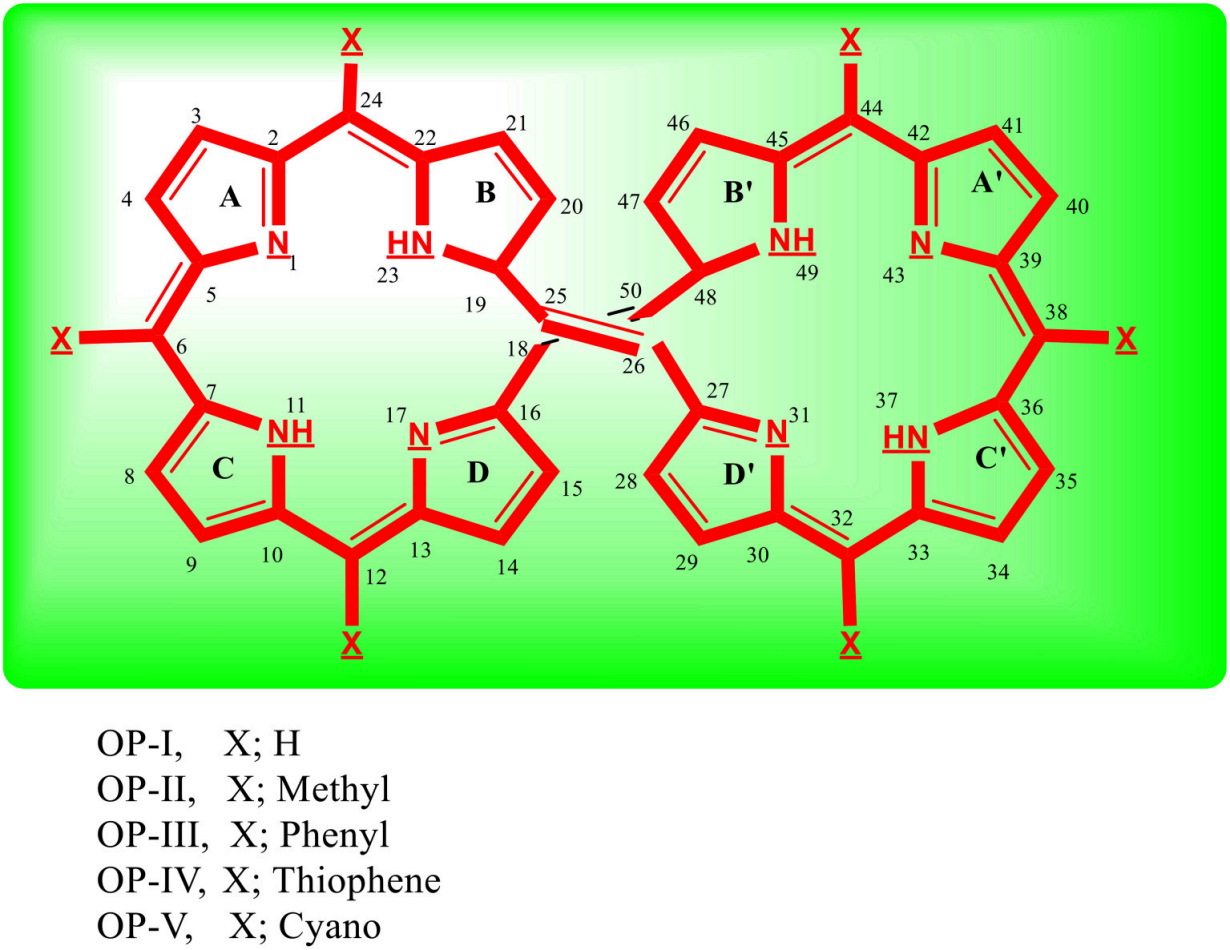
**Sketch of octaphyrin (OP) derivatives study using DFT at CAM-B3LYP/6-311++G (2d, 2p) level of theory**.

Conjugated organic systems are principally explored as hole transport materials for the organic light emitting devices (Fink et al., 1998; Jurchescu et al., 2004; Park et al., 2011; Tao et al., 2011; Kim et al., 2013; Wu et al., 2014; Islam and Pandith, 2014b; Fan et al., 2015). Because of troublesome processbility and instability in air, the exploration, design and synthesis of electron transport material has remained a significant challenge and hot stock within the field of organic electronics (Pandith and Islam, 2014; Zhao et al., 2014). Within the recent years, conjugated nitrogen containing organic derivatives have been found as promising n-type or p-type materials for the fabrication of OLED (Fink et al., 1998; Jurchescu et al., 2004; Park et al., 2011; Tao et al., 2011; Kim et al., 2013; Pandith and Islam, 2014; Wu et al., 2014; Zhao et al., 2014; Islam and Pandith, 2014b; Fan et al., 2015). The material acquires this property because of their excellent optoelectronic properties, good oxidation and thermal stabilities, large electron affinities and high electron mobilities. In the first section of paper, we have studied optoelectronic properties of octaphyrin derivatives (**OP**) (Figure 1) to explore their potential as n-type material for OLED devices and have calculated their reorganization energies, ionization potentials and electron affinity values. In the second section we have explored the octaphyrin derivatives as a class of organic molecules suitable for NLO applications.

A significant interest still exists for novel molecular materials with optimal NLO properties because of their primary roles in applications in fields including optical communications and computation, optical switching and limiting, data storage and retrieval, and sensors (Islam et al., 2014; Dadsetani and Omidi, 2015). The nonlinear response of molecules to electromagnetic fields has been studied over the last two decades. It provides a way for amplification, modulation and changing the frequency of optical signals (Liu et al., 2011; Lin et al., 2015). Materials with massive hyperpolarizabilities have found applications in optoelectronics, such as optical switching for optical computing or high-density optical recording. Several experimental and theoretical studies have been administrated to identify materials with large hyperpolarizabilities (Datta and Pati, 2006; Lakshmi et al., 2008; Lin et al., 2013; Rintoul et al., 2013; Dai et al., 2014; Liu et al., 2014; Islam and Chimni, 2017). As a result of distinctive structure octaphyrins with extended π electrons can prove smart candidates for NLO response. So as to visualize its optical properties, we have calculated the polarizability (α), the first-order hyperpolarizability (β) and also the second-order hyperpolarizability (γ**)** of octaphyrin. According to Dai et al. (2014); Rintoul et al. (2013); Lin et al. (2013); Liu et al. (2014), Datta and Pati (2006), Lakshmi et al. (2008) and Islam and Chimni (2017) second-order response governed by the second order hyperpolarizability offers more varied and richer behavior than first-order NLO process due to the higher dimensionality of the frequency space. Therefore, we have also calculated the second-order hyperpolarizability of the octaphyrin derivatives, in order to conclude their appropriate NLO response.

## Computational studies

In the present study, structural and optoelectronic calculations of **OP** derivatives were performed by using Density functional theory. The geometries were optimized by employing CAM-B3LYP (Yanai et al., 2004) functional using 6-31G (2d, 2p) (Ditchfield et al., 1971), and 6-311++G (2d, 2p) (McLean and Chandler, 1980) basis sets. Frequency calculations at the same level of theory were performed to confirm each stationary point to be a true energy minimum. The neutral molecules were treated as closed-shell systems, while for the radical anion or cation open-shell system optimizations were carried out using a spin unrestricted wave functional. The parameters governing the NLO properties calculated at CAM-B3LYP functional were found comparbale more accurate in the previous studies (Torrent-Sucarrat et al., 2011). The electronic absorption spectra of **OP** derivatives were calculated at TD-DFT/CAM-B3LYP/6-311++G (2d, 2p) level of theory. The calculation for the lowest transition was derived from the Gaussian output file using the GaussSum program (O'Boyle et al., 2008). All the calculations were performed using the Gaussian 09 computational package (Frisch et al., 2009).

## Results and discussion

**Figure 2:**
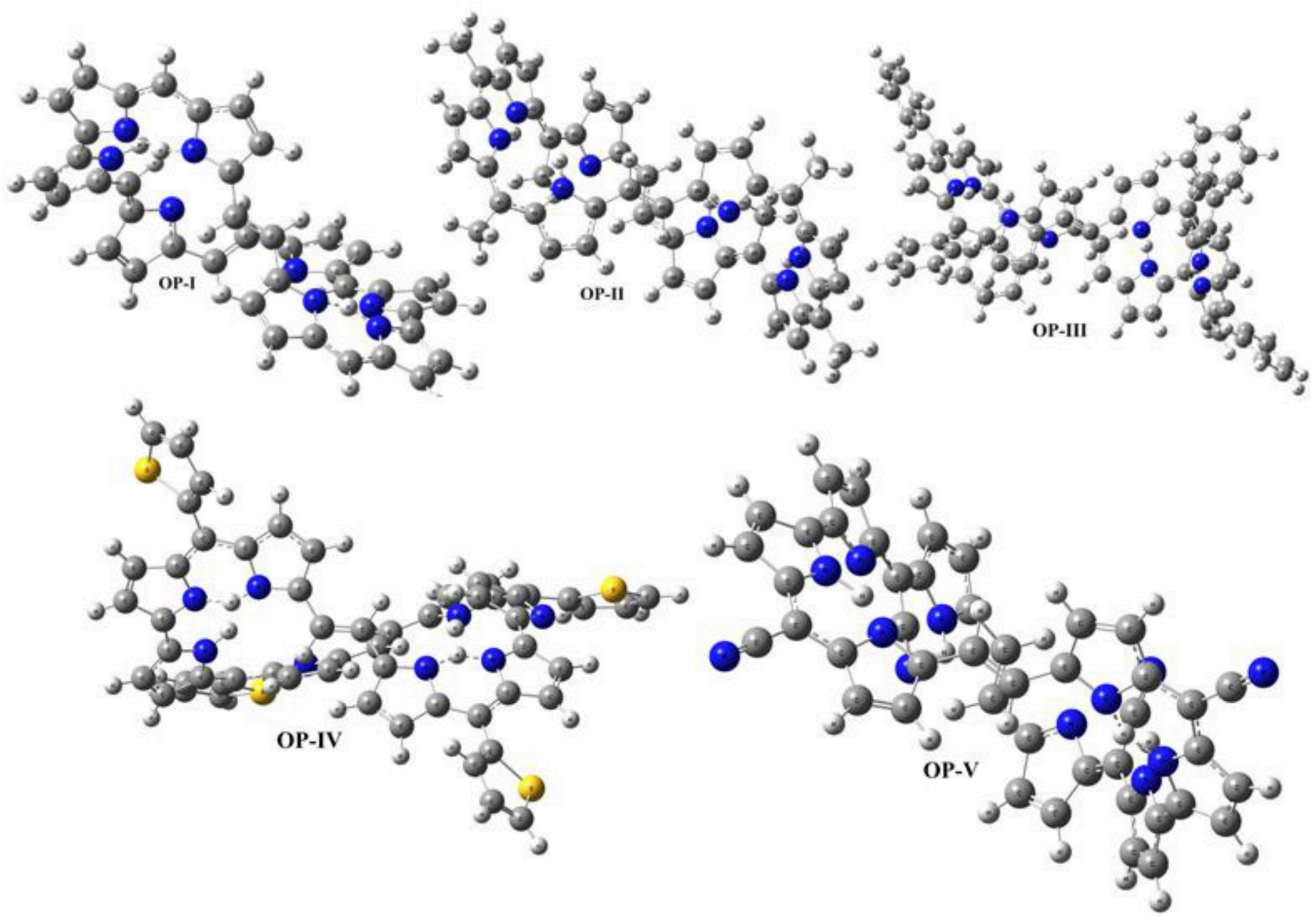
**Optimized geometries of OP derivatives obtained at CAM-B3LYP/6-311++G (2d, 2p) level of calculations**.

The optimized geometries of **OP** derivatives obtained from DFT calculations are illustrated in Figure 2. These systems exist in two atropisomeric forms ***P*** and ***M*** (Sprutta and Latos-Grazyński, 2001; Flemming and Dolphin, 2002; Pushpan et al., 2002; Silva et al., 2002a,b; Cavaleiro et al., 2003; Hata et al., 2006; Tanaka and Osuka, 2016). ***P*** and ***M*** isomeric forms are the mirrors images of each other, which do not show any distinction in electronic properties. The assignment of atropisomeric forms depends on the sense of the helical twist, it could be clockwise and denoted by **P** (“plus”) for a right-handed helix or be anticlockwise and denoted by **M** (“minus”) for a left-handed helix. In this study ***P***-isomer was considered for the evaluation of electronic and optical properties. Computational calculations display that all the studied **OP** geometries vary in the orientation of pyrrole rings and the two centers, each containing four pyrrole rings, are coplanar to each other. In **OP-I** pyrrole rings are oriented in such a manner that all the nitrogen groups are toward the center (core) of molecule. In case of **OP-II** and **OP-III** the methyl and the phenyl groups present on C_24_ and C_32_ are oriented toward the center (core) resulting in hyperbolic nonlinearity in the derivatives respectively. However, in case of **OP-IV** the pyrrole ring D and B are oriented away from the center, resulting in chair type geometry of the derivatives. The thiophene rings remain away from core and point toward the corner position of the three dimensional box in **OP-IV** derivative. Cyano group being linear, thus the geometry of **OP-V** does not vary abundantly from **OP-I**. In addition, cyano groups are directed alternatively away and toward the center of octaphyrin segment. Comparing the bond length of the **OP** derivatives, the C_16_-C_18_ bond is shorter in case of **OP-V** as compared to OP-I following the trend OP-V < OP-IV < OP-III < OP-II = OP-I. On the other hand the bond length of C_18_ = C_26_ within derivatives is longer just in case of **OP-V** as compared to **OP-I**. The decrease in the C_16_-C_18_ bond length shows some double bond character, which suggests that upon substitution with cyano group the π electron delocalization is enhanced over the entire frame work instead of localized between the particular nuclei. Thus, electron delocalization leads to the change in bond length or polarization in these systems. The impact of substitution on the aromaticity and charge transfer was analyzed by calculating the bond length alternation (**BLA**) values. Bond length alternation is a construct that can be used to monitor the amount of change in polarization across the bonds in a molecule upon substitution. Fu et al. (2008) defined BLA as the average of the difference in the length between adjacent carbon-carbon bonds in a polymethine [(CH)_*n*_] chain. In this work we calculated a local BLA associated with the C_16_-C_18_, C_18_ = C_26_ and C_26_-C_48_ bond lengths and consistent with the definition given by Fu et al., BLA is given as:

(1) $$\text{BLA}=\frac{\text{d}\left({\text{C}}_{\text{16}}-{\text{C}}_{\text{18}}\right)+\text{d}\left({\text{C}}_{\text{26}}-{\text{C}}_{\text{48}}\right)}{2}-\text{d}\left({\text{C}}_{\text{18}}={\text{C}}_{\text{26}}\right)$$

The BLA values obtained from the above equation follow the trend OP-V (0.261) < OP-III (0.297) < OP-II (0.306) < OP-IV (0.310) < OP-I (0.312). Thus, the substitution of hydrogen atom with electron withdrawing groups (cyano here) has less effect on the geometry of **OP**. The OP-I displays higher value followed by OP-II, OP-III and OP-IV indicating that the presence of methyl, phenyl and thiophene substituents have a scarce influence on the aromaticity of the series of studied compounds.

### OLED properties

The charge transfer properties play a vital role in high performance OLED devices. Charge transfer within the materials is often viewed in light of two headings, i.e., hopping theory (Lin et al., 2003) and the band theory (Cheng et al., 2003). Under the frame of hopping theory, the charge carrier is absolutely localized on a single molecule for the limit of thermal disorder and therefore the charge transfer happens between small coupled neighboring molecules. However, according to the band theory transport of charge is an activationless process, occurring through bands fashioned by the overlapping MOs between neighboring molecules. Under the heading of the hopping model the rate of charge transfer is described by the standard Marcus electron-transfer theory. According to Marcus the rate of electron or hole-transfer ***k***_*et*_ is given by the following equation (Marcus, 1957a,b).

(2)  ket= (4π2h )ΔHab2(4πλkBT)−12exp(−(ΔG0+λ)24λkBT  )  

were λ and Δ***H***_*ab*_ are the reorganization energy for the intramolecular electron transfer and the electronic coupling integral between donor-acceptor pair, respectively, and ΔG^0^ is the Gibbs free energy change of the process. The reorganization energy includes the contributions from the intramolecular and intermolecular energy change during a charge transfer event. The intramolecular reorganization energy refers to the relaxation of the molecule involved in the charge transfer process and the intermolecular reorganization energy refers to the relaxation of the medium in which the charge transfer takes place. From Equation (2) it is clear that λ should be low to get a high electron or hole transfer rate. Various studies have defined that first-principles quantum chemistry calculations would be productive to investigate the charge transport properties. In this study we have focused on estimating the intramolecular reorganization energy (λ) to evaluate the optoelectronic properties of studied molecule. So, the reorganization energies for the hole and electron transfers are evaluated using the following formulas (Tavernier and Fayer, 2000).

(3) $$\lambda_{h}=\left[E\left(M^{+}\right)\right]-\left[E\left(M\right)\right]+\left[E^{+}\left(M\right)-E^{+}\left(M^{+}\right)\right]$$

(4) $$\lambda_{e}=\left[E\left(M^{-}\right)\right]-\left[E\left(M\right)\right]+\left[E^{-}\left(M\right)-E^{-}\left(M^{-}\right)\right]$$

where E(M), E^+^(M^+^), and E^−^(M^−^) are the respective energies of optimized neutral, cationic, and anionic structures. E (M^+^)/E(M^−^) is the neutral energy of the optimized cationic/anionic structure, and E^+^(M)/E^−^(M)is the cationic/anionic energy of the optimized neutral structure. The calculated values of intramolecular reorganization energies for **OP** derivatives are given in Table 1. We have observed that λ_*e*_ (reorganization energy for electron transport) values are comparatively smaller as compared to the λ_*h*_ (reorganization energy for hole-transport) values demonstrating relevance of studied **OP** derivatives as n-type material for Organic Light Emitting devices. However, upon derivatization the value of λ_*e*_ further decreases and is a minimum for **OP-V** containing CN-groups. The λ_*e*_ values obtained for OP derivatives were found smaller as compared to reported compounds projected as economical n-type candidates for OLED devices (Pandith and Islam, 2014; Zhao et al., 2014; Naka et al., 2000). According to Liu et al. (2010) the ionization potential (IP) together with the electron affinity (EA) can be used to weigh the hole and electron injection properties respectively. The vertical and adiabatic ionization potential and electronic affinity of OP derivates were calculated by using the following equations and are given in Table 1.

(5) $$IP(v)/IP\left(a\right)=\;E^{+}\left(M\right)/E^{+}\left(M^{+}\right)-E\left(M\right)$$

(6) $$EA(v)/EA\left(a\right)=\;E\left(M\right)\;-E^{-}\left(M\right)/E^{-}\left(M^{-}\right)$$

**Table 1:** **Ionization energy, adiabatic energy, and reorganization energy of OP derivatives calculated by employing DFT/CAM-B3LYP/6-311++G (2d, 2p) level of theory**.

	**IP_adiabatic_ (eV)**	**IP_vertical_ (eV)**	**EA_adiabatic_ (eV)**	**EA_vertical_ (eV)**	**λ_hole_ (eV)**	**λ_electron_ (eV)**
OP-I	6.82	6.89	3.09	3.13	0.211	0.131
OP-II	6.87	6.93	3.32	3.33	0.209	0.129
OP-III	6.92	6.96	3.41	3.49	0.221	0.124
OP-IV	6.98	7.01	3.53	3.51	0.228	0.121
OP-V	7.04	7.12	3.61	3.63	0.217	0.118

*Were IP_adiabatic_ is adiabatic ionization potential, IP_vertical_ vertical ionization potential, EA_adiabatic_ adiabatic electron affinity, EA_vertical_ vertical electron affinity, λ_hole_ and λ_electron_ reorganization energy for hole and electron transport respectively*.

**Figure 3:**
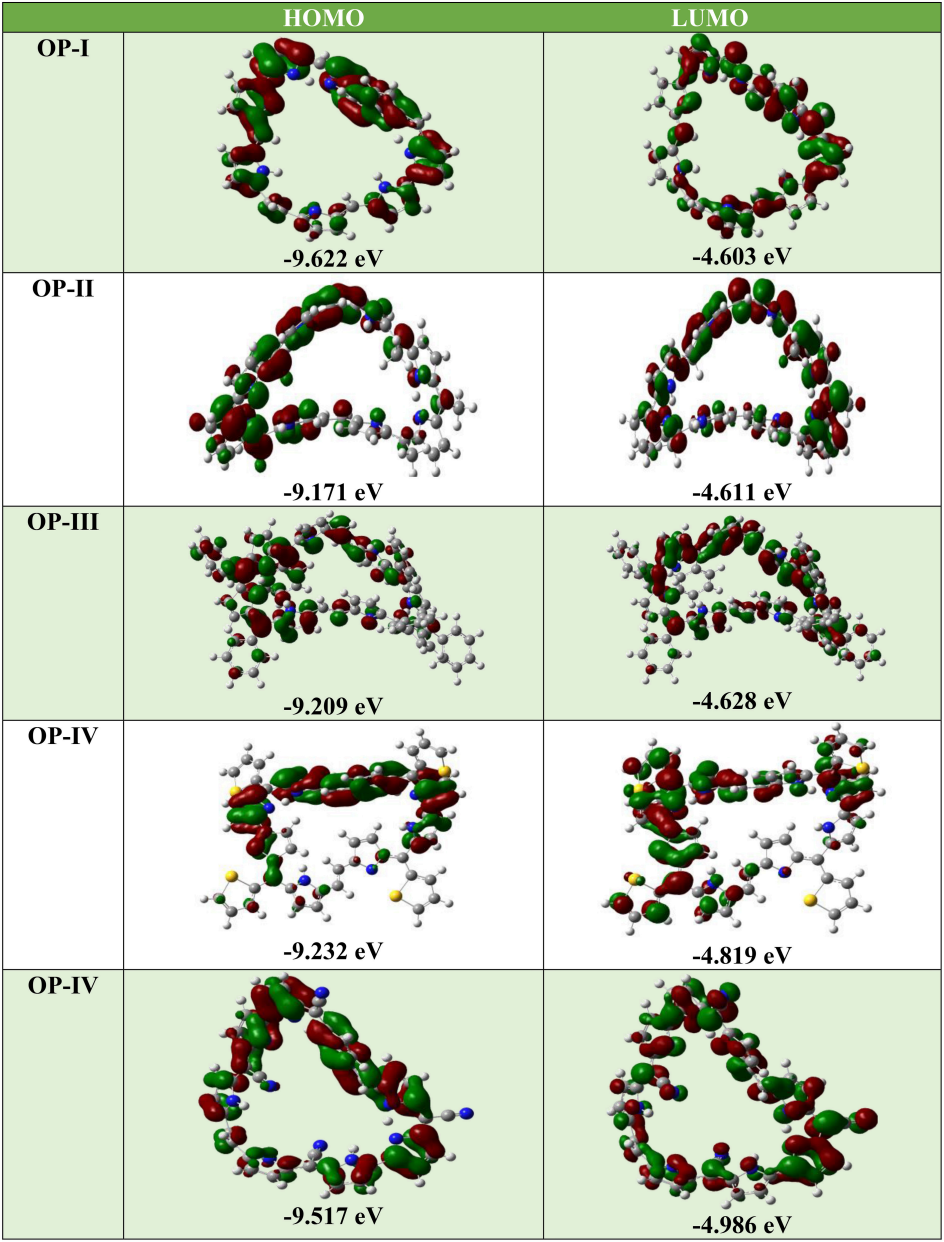
**The frontier molecular orbitals (FMOs) of OP derivatives at DFT/ CAM-B3LYP/6-311++G (2d, 2p) level of theory**.

Where *IP(v)*/*IP(a)* and *EA(v)*/*EA(a)* are vertical and adiabatic ionization potential and electron affinity respectively. The calculated EA values for the OP derivatives are all greater than 3.00 eV defining their anionic stability toward the oxygen present in surrounding. Based on the recent theoretical studies EA for air stable n-type material should be greater than 2.80 eV (Newman et al., 2004; Chang et al., 2010). Thus, according to these observations the studied compounds are quite stable to moisture present in air as n-type material on account of their large adiabatic electron affinity values. On the basis of above calculations, the electron affinity values of OP derivatives follow the trend OP-I < OP-II < OP-III < OP-IV < OP-V (Table 1). The OP-V scores maximum EA and IP value; therefore in case of OP-V the holes and electrons can be consequently injected into the emissive layer much more easily. Thus, combining the relationships between charge injection and the values of EA, it is concluded that the electron injection properties are improved with the introduction of electron withdrawing groups. All the studied octaphyrins exhibit π-character that spreads over the entire molecule resulting in delocalization, demonstrating their efficient charge transfer ability. The theoretical calculation shows that these molecules have low lying HOMO and LUMO energy levels that signifies that they possess high oxidation and reduction stabilities. As seen from Table 2 the LUMO of the OP derivatives reaches from −4.6 to −4.9 with the substitution. On relating our findings with the calculations carried by Usta et al., the studied OP can be applicable with the work function of the metal electrode like Au (−5.1 eV) and Pt (−5.6 eV) used in practical OFET devices (Usta et al., 2009). It has been observed that the electron injection potential barrier for n-type material between the OP and metal electrode decreases with the introduction of an electron withdrawing group on OP skeleton. The frontier molecular orbital analysis (Figure 3) displays the change in HOMO-LUMO distribution over the OP framework initiated either by mesomeric or inductive effect. The former has relevance to the sharing of π-electrons between the parent and substituents whereas the latter is expounded to the σ–electron systems.

**Table 2:** **FMO (HOMO and LUMO energies) and Optical data of OP derivatives calculated by employing DFT and TDFT level of theory respectively**.

	**HOMO (eV)**	**LUMO (eV)**	**ΔE**	**λ_*max*_**	**E_*ge*_**	**f**	**Orbital contribution**
OP-I	−9.2622	−4.603	4.659	530	4.341	0.722	HOMO → LUMO 81 %
OP-II	−9.1713	−4.611	4.560	538	4.217	0.718	HOMO → LUMO 69 %
OP-III	−9.2099	−4.628	4.582	540	4.202	0.701	HOMO → LUMO 78 %
OP-IV	−9.2326	−4.819	4.414	612	4.001	1.015	HOMO → LUMO 95 %
OP-V	−9.5175	−4.986	4.532	526	4.112	0.855	HOMO → LUMO 90 %

*Were HOMO is highest occupied molecular orbital energy, LUMO lowest unoccupied molecular orbital energy, ΔE energy gap between HOMO and LUMO, λ_max_ maximum absorption wavelength, E_ge_ excitation energy and f oscillator strength corresponding to S_0_ to S_1_ excitation*.

For the above system the LUMO energies decrease both with electron donating as well as electron withdrawing substituents and attributable to different decreasing degrees in HOMO and LUMO the energy gaps for these OP derivatives become distinguishable. Analyzing the energy gap between the HOMO and LUMO, the **OP-IV** displays the lowest value and **OP-I** the highest value. Thus, the potency of OP derivatives as n-type material as per the values of EA and λ_*e*_ does not correlate well with the energy gap between HOMO and LUMO. The frontier molecular orbital (FMO) studies reveal that the highest occupied molecular orbitals (HOMOs) of the neutral molecules delocalize primarily over the octaphyrin skeleton with least contribution from the substituents, whereas the lowest unoccupied molecular orbitals (LUMOs) have an obvious electron density distribution including contribution from Octaphyrin skeleton and as well as from substituents. These orbital pictures defines that the energy levels of the LUMOs rather than that of HOMOs are more affected by the introduction of the substituent moiety. The TD-DFT studies at CAM-B3LYP/6-311++G (2d, 2p) level of theory reveal that in all octaphyrin moieties the absorption spectra display a single peak with slight red shift in case of cyano group and remarkable blue shift in case of OP-IV. From Table 2, the primary excited state (S1) of **OP** derivatives originates from the HOMO to LUMO transitions with contributions of about 95 and 69% in case OP-IV and OP-II respectively. Thus, on the basis of above calculations we will conclude that OPs sustain position as an optoelectronic material, however, by introducing electron withdrawing groups they will prove as an effective n-type material for OLEDs.

### Nonlinear optical response

Octaphyrin being a conjugated pathway can become a potential material for NLO response. NLO techniques are considered as among the most structure-sensitive methods to study the molecular structures and assemblies. Quantum chemical calculations are shown to be useful within the description of the relationship among the electronic structure of these systems and their NLO response. During this study, the dipole moment, polarizability and first-order hyperpolarizability and second order hyperpolarizability were used to evaluate the nonlinear response of Octaphyrin derivatives. The average linear polarizability <α>, first order hyperpolarizability < β > and second order hyperpolarizability < γ> values have been calculated from Gaussian output file using the following relations.

The microscopic polarizability (P) induced in an isolated molecule under the applied electric field (E) of an incident electromagnetic wave can be expressed by the following equation:

(7) $$P=\;\alpha_{E}\;+\;\beta_{EE}$$

Where P and E are related to the tensor quantities α and β which are referred to as the polarizability and hyperpolarizability, respectively.

The definition (Sajan et al., 2006; Alyar et al., 2007; Sundaraganesan et al., 2009; Zhang et al., 2010) for the polarizability is:

(8) $$\langle \alpha \rangle ={\;}^{1}{╱}_{3}\;\;\left(\alpha_{xx}+\;\alpha_{yy}+\alpha_{zz}\right)$$

The anisotropy of polarizability is:

(9) $$\begin{array}{l} \Delta \alpha =1/\surd 2[{\left(\alpha_{xx}-\alpha_{yy}\right)}^{2}+{\left(\alpha_{yy}-\alpha_{zz}\right)}^{2}+{\left(\alpha_{zz}-\alpha_{xx}\right)}^{2}\\ \;+{6\alpha_{xz}^{2}\;+\;6\alpha_{xy}^{2}+6\alpha_{yz}^{2}]}^{½} \end{array}$$

And the first hyperpolarizability is a third rank tensor that can be described by a 3 × 3 × 3 matrix (Thanthiriwatte and de Silva, 2002). The components of can β be calculated using the following equation:

(10) $$\begin{array}{l} \langle \beta \rangle =\;[{\left(\beta_{xxx}+\beta_{xyy}+\beta_{xzz}\;\right)}^{2}+\;{\left(\beta_{yyy}+\beta_{yzz}+\beta_{yxx}\;\right)}^{2}\\ \;{+\;{\left(\beta_{zzz}+\beta_{zxx}+\beta_{xyy}\;\right)}^{2}]}^{½} \end{array}$$

or simply:

(11) $$\beta_{∥}\;=\;\frac{1}{5}\;\sum_{i}\left(\beta_{iiz}+\beta_{izi}+\beta_{zii}\right)\;(i\;from\;x\;to\;z)$$

and average value of second order hyperpolarizability (Kurtz et al., 1990) is:

(12) $$<\gamma >\;=\;\frac{1}{5}\;\left[\gamma_{xxxx}+\gamma_{yyyy}+\gamma_{zzzz}+\;2\left[\gamma_{xxyy}+\gamma_{yyzz}+\gamma_{xxzz}\right]\right]$$

From Table 3 the changes in the NLO properties were observed after the introduction of different electron donating and withdrawing groups into **OP** framework. For the neutral forms I–V, β and γ values decrease in the order IV > III > V > II > I. For the assorted R groups, the thiophene group is a strong electron-donating group and the cyano is a strong electron withdrawing group and from the values in Table 3 it is concluded that the introduction of a strong electron-donating group into OP is favorable for improving NLO responses. This is attributed to increase in electron density of the OP framework resulting in enhancement in induced ring current. However, introducing an electron withdrawing group like CN, enhances the NLO properties still as compared to OP-I but to a lesser extent. This unexpected NLO response of OP-V implies that the charge transfer pattern of OP-V is polydirectional in comparison with the OP-I. The calculated values of α and β for the studied OP dertivative were found close to Bianthraquinodimethane Modified [16]Annulene (Torrent-Sucarrat et al., 2011). So as to achieve more insight into NLO response of OP derivatives, we correlated two level model with first and second order hyperpolarizability of these derivatives. Oudar and Chemla by employing complex sum over states (SOS) expression established a simple link between β and low-lying charge-transfer transition through the two-level model (Oudar and Chemla, 1977). According to this model the static first hyperpolarizability is expressed by the following expression:

(13) $$\beta \;\propto \left(\mu_{ee}-\;\mu_{gg}\;\right)\;\frac{\mu_{ge}^{2}}{E_{ge}^{2}}$$

where μ_*ee*_ and μ_*gg*_ are the ground state and excited-state dipole moment, μ_*ge*_ is the transition dipole, and *E*_*ge*_ is the transition energy.

**Table 3:** **DFT/TDFT calculated average values of the dipole moment (in ground (μ_*gg*_) and excited state (μ_*ee*_), transitions dipole moment ($\mu_{ge}^{2}$, static polarizability α (Å^3^), the first polarizability β (esu.) and the second polarizability γ (esu) for OP derivatives**.

	**μ_*gg*_**	**α (× 10^−23^)**	**β (× 10^−33^)**	**γ (× 10^−35^)**	**μ_*ee*_**	**$\mu_{ge}^{2}$**	**μge2Ege2**
OP-I	3.80	93.98	2,086.31	−3353.42	3.92	0.94	0.048
OP-II	4.54	144.29	7,906.63	−2196.50	4.69	0.99	0.056
OP-III	4.91	157.20	7,911.70	−1686.21	5.18	1.01	0.057
OP-IV	5.37	203.23	10,349.22	445.33	6.81	1.15	0.072
OP-V	3.98	111.50	4,038.53	−2122.97	4.51	1.08	0.063

As seen in Table 3 the improvement in β values with increasing electron donating abilities can be attributed to the increasing μ_*ge*_ and decreasing *E*_*ge*_ values. The result shows that the μge2Ege2 values of OP derivatives follow the trend IV > V > III > II > 1 and is in agreement with the β as well as γ values except in case of OP-V. On comparing the OP-V (cyano substituted molecule) with OP-III (phenyl substituted molecules), it is found that OP-III compound possess smaller $\mu_{ge}^{2}$ value. However, the static hyperpolarizability of the **OP-III** is higher than that of the cyano substituted molecule. The second order hyperpolarizabilities of **OP-II** and **OP-V** do not differ much instead of huge differences in their first order hyperpolarizabilities. Additionally, there is a good correlation between the hyperpolarizabilities and the BLA values on changing the donor groups. The trend for the hyperpolarizabilities and BLA values is OP-I < OP-II < OP-III < OP-V < OP-IV and OP-I > OP-II > OP-III < OP-V < OP-IV respectively. Thus, the acceptor group has little effect on the BLA value and does not contribute much to the change in hyperpolarizabilities. However, changing the donor groups has a better effect on the BLA value and the hyperpolarizabilities of these systems. Thus, our investigation has shown that substitution with electron withdrawing group like cyano enhances the n-type ability of **OP**, however the substitution with electron donating group like thiophene multiplies the NLO response of octaphyrins.

## Conclusions

The charge transport and nonlinear performance of octaphyrin derivatives have been studied using DFT level of theory. Computationally calculated properties like electron affinity and ionization energy values show that all these derivatives are stable toward oxygen and water in the air. The reorganization energy values show that these octaphyrin derivatives are effective as n -type materials. The low lying orbital energy levels signify that OP derivatives show high oxidation and reduction stabilities. Nonlinear response of these derivatives was quantified in terms of polarizability and hyperpolarizability values. Understanding the above carried computational studies, it is concluded that the n-type material character enhances by introduction of electron withdrawing groups but the nonlinear response is solely increased by electron donating groups.

## Author contributions

The work is designed and done by NI. IL assists in doing the calculations.

### Conflict of interest statement

The authors declare that the research was conducted in the absence of any commercial or financial relationships that could be construed as a potential conflict of interest.
